# Belowground eco-restoration of a suburban waste-storage landscape: Earthworm dynamics in grassland and in a succession of woody vegetation covers^☆^

**DOI:** 10.1016/j.landurbplan.2013.06.007

**Published:** 2013-12

**Authors:** Pamela K. Morales, Isa A.M. Yunusa, Glenys Lugg, Zheng Li, Paul Gribben, Derek Eamus

**Affiliations:** a Plant Functional Biology and Climate Change Cluster, University of Technology, Broadway, Sydney, NSW 2007, Australia; b School of Environmental and Rural Sciences, University of New England, Armidale, NSW 2350, Australia; c Manildra Group, 36 Bolong Road, Bomaderry, NSW 2541, Australia

**Keywords:** Earthworms, Belowground ecological restoration, Soil nutrient content, Soil salinity, Vegetation age, Waste management

## Abstract

Restoration of belowground ecology is seldom a priority in designing revegetation strategies for disturbed landscapes. We determined earthworm abundance and diversity in a 16-year old grass sward (grassland), a 6-year old (Plantation-04) and a 4-year old (Plantation-06) plantation, both of mixed woody species, on a reclaimed waste disposal site, and in nearby remnant woodland, in suburban Sydney, Australia. While no catches were made in autumn, more earthworms were found in spring (21 ± 8.6 m^–2^) than in winter (10.2 ± 5.9 m^–2^) or summer (14.4 ± 5.5 m^–2^). Earthworm abundance in spring was in the order grassland ≈ Plantation-04 (35.2 m^–2^) > woodland (12.8 m^–2^) > Plantation-06 (0.8 m^–2^). None of the revegetated covers had restored earthworm diversity to levels found in the woodland. Exotic species, mostly *Microscolex dubius*, dominated in the four vegetation covers at any time; the only two native species (*Heteroporodrilus* sp. and *Megascoleceides* sp.) found were in the woodland. We also assessed how quality of the evolving soils from the three revegetated covers, compared with that from the woodland, impacted viability of common exotic earthworm species. Both weight gain and cocoon production by the exotic earthworms were higher in the soil from Plantation-04 than in soils from the other vegetation covers, including the woodland; the two variables were positively correlated with the pH and mineral nutrient content (as indicated by electrical conductivity that was in turn correlated with clay content) of the soil. Age of vegetation rather than its composition explained differences in the level of earthworm recovery observed.

## Highlights

• Earthworms were most abundant and diverse in summer.
• Evaluate success of belowground eco-restoration in spring/summer season.
• Age of vegetation more critical than type for belowground eco-restoration.
• Belowground eco-restoration most effective in woody/herbaceous mixtures.
• Woody/herbaceous mixtures also have hydrologic and aesthetic benefits.

## 1 Introduction

There is an over-reliance on aboveground vegetation processes, including plant growth, in assessing efficacy of revegetation strategies for landscapes used for waste disposal or mining. This is because restoration of ecohydrologic process to minimize the risk of chemical and particulate pollution of the atmospheric and water resources often takes priority over other considerations. Restoration of soil ecosystem tends to receive little attention, despite its central role in maintaining soil health that ultimately underpins success of the planted vegetation and its dependent processes (Bradshaw, 1984). Both the chemical and physical properties of the recovered soil, along with its reprocessing and use in seedbed preparation, all impact on the survival and viability of the macrofauna, and on that of the plant species.

The need to conserve local biodiversity and aesthetics has increased the use of native plant species for ecological restoration (Grant, Campbell, & Charnock, 2002; Weir, Fulton, & Menzies, 2006). Woody species are therefore the appropriate choice for environments along the coastal fringes of southeastern Australia, where woodland is the dominant natural vegetation. However, relatively high cost and the long lead-time to achieve effective vegetation cover with woody species, make the use of herbaceous species, grasses in particular, attractive in some instances (Richardson, Burn, & Craig, 1987). Differences in species composition of vegetation covers can alter the evolving belowground ecosystems on rehabilitated landscapes. For instance restoration of physical properties, such as porosity and permeability, may take longer under grassland than woodland (Richardson et al., 1987; Yunusa et al., 2012), and will have significant influence on the developing soil biota such as earthworm communities (Chan and Barchia, 2007) or ants (Gollan, Lobry de Bruyn, Reid, Smith, & Wilkie, 2010).

Earthworms are effective indicators of soil health because of their sensitivity to both immutable and anthropogenic stresses in the soil, and are widely used to assess the ecological consequences of disturbance arising from changes in land use (Boyer and Wratten, 2010; Chan and Heenan, 2006; Hendrix et al., 2006; Smith et al., 2008). For instance, Smith et al. (2008) found a strong tendency towards increasing earthworm populations (abundance) in minimally disturbed landscapes, with earthworms being most abundant in an old growth forest. In addition to changes in abundance, land disturbance also induces strong alterations in the distribution of earthworm species. Land disturbance often results in the displacement of native earthworm species by exotic species (Chan and Heenan, 2006) such as the European *Aporrectodea* species that tend to easily displace pre-existing native species on disturbed lands (Scullion, Ramshaw, & Mohammed, 1988; Smith et al., 2008). It is probable that reconstituted soils may become inhospitable to native species, and in Australia exotic species are known to thrive better than native species in soils contaminated with heavymetals (Yunusa, Braun, & Lawrie, 2009) by minimizing ingestion of potentially harmful trace metals (Muir et al., 2007). The rarity of native species in disturbed lands could, however, be simply a function of time needed to recolonize the now changed habitat and readjust to the now changed edaphic environment, a processes that may take decades (Boyer and Wratten, 2010; Bradshaw, 2000).

In this study we used earthworm abundance, as surrogate of the whole soil biota, to assess the degree to which three vegetation covers consisting of a 16-year old grassland sward and a four- and a six-year old woody vegetation covers had restored earthworm communities on a reclaimed waste disposal site, when compared with an old growth woodland. We undertook field surveys to characterize seasonal changes in the abundance and diversity of earthworms in the four vegetation covers, and laboratory experiments to test how the soils from the four vegetation covers impacted viability of two common exotic earthworm species.

## 2 Materials and methods

This study was conducted at the Waste Management Centre at Castlereagh (33°39′41″S, 150°46′57″E or Google Locator −33.658781,150.780973) approximately 65 km north-west of central Sydney. The site covers an area of 357 hectares and the original soil at the site is classified as Chromosol, which is equivalent to Haplic Xerosol (FAO, 1974). This soil has a duplex profile consisting of 0.7 m loamy sand topsoil over impermeable heavy clay referred to as Londonderry Clay overlaying conglomerate sandstone and shales of the Triassic Wianamatta Group (Itakura, Airey, & Dobrolot, 2005). Storage cells consisting of trenches (20 m × 5 m, and 5 m deep) constructed into the clay subsoil, and spaced 2 m apart resulting in approximately 65 cells/ha. Once filled with wastes, the cells were capped using the excavated soil that was returned in reverse order of their removal. The cap forms a profile of 2 m over the cells. The reconstructed soil was then planted with either grasses or mixtures of woody and herbaceous species. For this project, we selected three vegetation covers along with nearby remnant woodland, described below:

*Grass pasture*: This was established in 1994 with a mixture of *Cynodon dactylon* (couch grassland), *Axonopus affinis* (carpet grassland), *Paspalum dilatatum* (paspalum), *Pennisetum clandestinum* and *Trifolium repens* (white clover). Prior to sowing, the soil was fertilized with 300 kg/ha or 30 mg/m^2^ of compound fertiliser containing mainly nitrogen, phosphorus and potassium (24:6:12). The grassland was about 16 years old at the start of this study, and it represented a relatively quick and cheap rehabilitation strategy.

*Plantation-04*: This was established in autumn (April–May) 2004 using a mixture of native trees and shrubs planted in 5 m rows. The tree species used were *Eucalyptus* spp., *Angophora* spp., *Casuarina glauca*, *Melaleuca linariifolia* and *Syncarpia glommulifera* and were interplanted with rows of shrubs made up of species of *Acacia*, *Callistemon*, *Grevillea*, *Hakea*, *Kunzea* and *Leptospermum*. Mineral fertiliser containing nitrogen, phosphorus and potassium (NPK) was applied at the time of establishment. The vegetation was almost six years old at the start of this study and provided a mixture of woody and herbaceous species as a mimic of native woodlands.

*Plantation-06*: This was established in 2006 using a mixture of woody species as in Plantation-04, but without shrub species or grassland groundcover. Herbaceous weedy species became established as groundcover. A thin layer (<0.1 m thick) of compost was sprayed over the soil in 2008, and was well incorporated into the topsoil at the time of this study. This cover represented a minimum rehabilitation strategy and was four years old at the start of this study.

*Woodland*: This is dominated by trees of *Eucalyptus parramentensis* and *Angophora bakeri*, in which the understorey is dominated by shrubs and grasslandes including *Pultinea elliptica* Smith, *Cryptandra amara* Smith and *Melaleuca thymifolia* (Yunusa et al., 2010). There was no record of any major disturbance of this vegetation aside from occasional fires, the last of which was in 1999.

All the four vegetation covers were within a 1.5 km radius of each other and have been described in detail in previous studies (Yunusa et al., 2010, 2012). Basic characteristics of the topsoil under the four vegetation covers are given in Table 1. The woodland is characterized by coarse sandy texture that is mildly acidic and saline with 0.044 dS m^−1^ and except for its higher total carbon content, had lower clay content and cation exchange capacity (CEC), and was less saline and dense, than those under the three revegetated sites all of which contained more trace metals (Table 1).

### 2.1 Field surveys

Field surveys were conducted on April 21, July 14, September 7 and December 15, 2010 to determine species diversity and abundance of earthworm communities under each of the vegetation covers. On each occasion, 20 quadrats (0.25 m × 0.25 m) were randomly sampled across each of the four vegetation covers. For each quadrat, the soil was collected to a depth of 0.25 m, sieved and the earthworms recovered which were then counted and transferred into bottles containing 70% ethanol. The earthworms were later sorted and identified in the laboratory after Blakemore (2008), although some juvenile earthworms could not be identified because of the incomplete development of their distinguishing features. The number of earthworms was expressed as mean population density and the percentage of adults was also determined.

Daily weather variables consisting of minimum and maximum temperature, humidity and rainfall for the site were monitored with an automatic weather station. These data were used along with canopy characteristics for the vegetation covers (Yunusa et al., 2012) to predict temperature at 0.2 m depth under the four vegetation covers using the model of Paul et al. (2004).

### 2.2 Laboratory experiments

A series of experiments were undertaken using soil samples collected from the four vegetation types. The soil samples from each of the vegetation type were combined and later mixed thoroughly in a cement mixer and to minimize effect of spatial variability during collection from the field; the composite samples were used to study how textural and basic chemical characteristics of these soils influenced survival, growth and activity of exotic and native earthworm species. The soil samples were ground and used to fill 1.5 L (top internal diameter 140 mm) pots. The pots were lined at the base with fly screen mesh (2.0 mm × 2.0 mm) and 20 mm thick coconut fibres to hold the soil and prevent any earthworm escapes. These pots were used in the following experiments.

#### 2.2.1 Survival and growth of exotic earthworms in soils from the four vegetation covers

Earthworms of *Aporrectodea trapezoides* or *Aporrectodea caliginosa* were each used to inoculate 64 pots; this allowed sampling of four replicates for each treatment at four time intervals. The pots were then thoroughly wetted with 1 L of deionised water two days before each was inoculated with three juvenile earthworms on the June 7, 2010. Average weight for a single worm was 0.66 ± 0.08 g for *A. trapezoides* and 0.40 ± 0.05 g for *A. caliginosa*. Prior to inoculation, the earthworms were washed with deionised water and then weighed before being placed in the centre of the pot and allowed to burrow. The earthworms were observed to burrow into the soils and disappeared within 5 minutes. The pots were then covered with fly screen mesh (3.0 mm × 3.0 mm) to prevent any escape through the soil surface.

After inoculation, the pots were randomly assigned to numbered spaces on a shelf in a temperature controlled growth room, which was kept at a constant temperature of 19 °C throughout the experiment. The pots were kept moist by adding 100 ml of deionised water every week. All the pots were supplied with 2 g of dry sheep manure at the start of the experiment, which was then administered every 2–3 weeks. The total amount of food administered to all pots by the end of the experiment was 10 g. At 2, 5, 8 and 11 weeks after inoculation, 32 pots made up of four each for *A. trapezoides* and *A. caliginosa* were randomly sampled from each soil type. At each sampling, the number and weight of surviving earthworms were determined, while the cocoons and casts were recovered from the surface of each pot and weighed following Muir et al. (2007). Soil in each pot was sampled to determine pH and electrical conductivity (EC).

#### 2.2.2 Assessment of textural and nutritional alterations of soil on survival and growth of exotic earthworms

Two experiments were undertaken to assess how alterations in the texture of the clayey soil from Plantation-04, and in the nutrient content of the loamy soil from the woodland, influence the growth of exotic endogeic *A. trapezoides* and *Amynthas gracilis* over four weeks from July 30 to the August 23, 2010.

The first experiment involved textural alteration of the clay soil in Plantation-04 using the following treatments:

i. Control: unamended soil.
ii. Sand amendment: 9 kg of washed sand was mixed with 9 kg of Plantation-04 soil.
iii. Organic matter amendment: soil mixed with 3.7% (w/w) of compost to raise the carbon content to 0.96%, close to the level found in the woodland.
iv. Sand and organic matter amendment: mixture of equal amounts of sand with 3.7% (w/w) of compost.

The mixtures were achieved with a cement mixer and then used to fill the pots as described above. The aim of the sand amendment was to alter the texture of the plantation soil by increasing the percentage of sand by up to 50%. Thirty two pots were set up in total and each inoculated with juvenile earthworms of either *A. trapezoides* or *A. gracilis* as described above, average weight for *A. trapezoides* was 0.52 ± 0.07 g and for *A. gracilis* was 0.89 ± 0.06 g. The response variables measured were: number and weight of surviving earthworms, number of cocoons and weight of casts. Soil sample from each pot was collected to determine pH and the EC.

The second experiment assessed how nutrient addition to the woodland soil influenced performance of exotic earthworms consisting of *A. caliginosa* and *A. trapezoides*. There were two treatments:

i. Control: unamended soil.
ii. Fertilizer amendment: a compound fertilizer containing nitrogen, phosphorus and potassium (15% N: 4.4% P: 10% K) at a rate equivalent to 300 kg/ha was mixed into the soil. This was achieved by mixing 17.3 g of fertiliser with 37 kg of soil, i.e. 0.047% (w/w), in the cement mixer.

These two soil treatments were used along with the two exotic earthworm species (*A. caliginosa* and *A. gracilis*) in a factorial design of four treatments with four replications, thus 16 pots were involved. Juvenile earthworms were used with mean weight of 0.25 g for *A. caliginosa* and 0.51 for *A. trapezoides*. The pots were maintained in the same manner as described above. Four weeks after inoculation, the earthworms were recovered and the following variables were measured: number and weight of earthworms, number of cocoons and weight of castings produced. Soil samples were also taken from each pot to determine pH and EC.

### 2.3 Data analysis

Statistical analysis of the data was carried out using SPSS v17 in which all data were tested for normality, while Levene's test was used to determine equality of variances. Statistical significance was determined when *p* ≤ 0.05. Tukeys HSD was used to test for differences between groups when the effect was significant. For the field survey data, one-way analysis of variance (ANOVA) was used to tests for differences in earthworm abundance between the four vegetation types. The extent to which earthworm population was restored by the three revegetation strategies was determined using the worm population collected in spring to calculate the effect size (*d*) after Gurevitch and Hedges (1993):

(1) $$d=\frac{{\bar{X}}_{\text{r}}-{\bar{X}}_{\text{w}}}{\sigma_{\text{pooted}}}$$

in which ${\bar{X}}_{\text{r}}$ and ${\bar{X}}_{\text{w}}$ are the respective mean values for the revegetated blocks (grassland, Plantation-06 or Plantation-04) and the woodland, and *σ*_pooled_ is the standard deviation for the pooled data for all the four vegetation covers. For the laboratory experiments, treatment effects were tested using two-way ANOVA.

## 3 Results

### 3.1 Seasonal trends in earthworm population composition

**Fig. 1:**
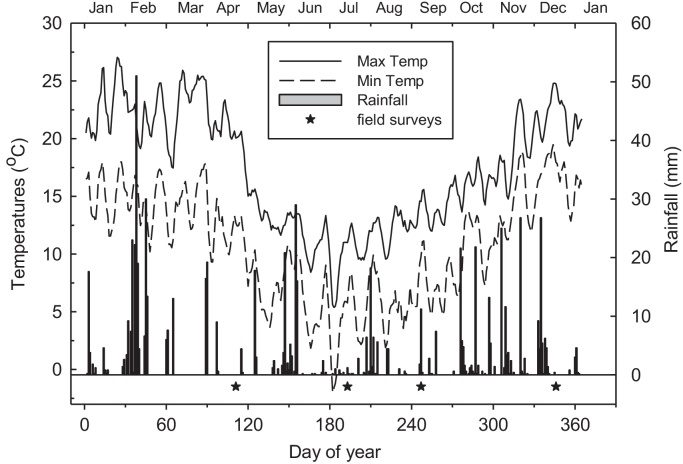
Mean weather conditions at Castlereagh, Australia, during the study in 2010. The stars indicate when field samplings were undertaken.

Weather conditions during the year were consistent with the sub-temperate climate of the site. The first sampling in April (autumn) followed almost a 2-month period when little rain fell (Fig. 1), while it was cool and relatively dry in July (winter). At the third in September (spring) the weather had warmed up and also wet due to frequent rainfalls, and this conditions largely persisted to the fourth sampling in December (summer). Model predictions showed that the soil was warmest in autumn and coldest in winter, with the difference being up to 5 °C (Table 2). The soil was always cooler under Plantation-04 and warmest in the grassland, where the soil temperature was often within 1 °C that in Plantation-06.

**Fig. 2:**
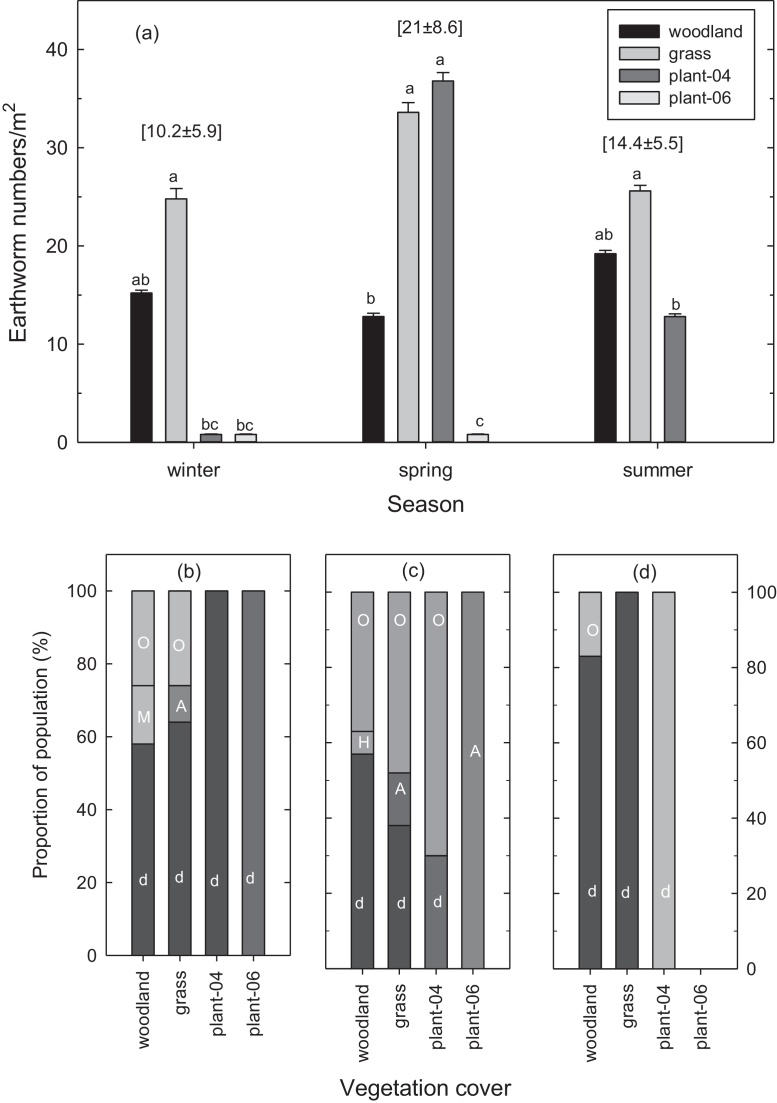
Population profiles for earthworms in 2010 at Castlereagh, Australia 2010: mean abundance of earthworms collected from the four vegetation covers during the three seasons (a), and species composition of the samples collected in winter (b), spring (c), and summer (d). In (a) numerals in [parentheses] are the mean (±standard error) of earthworms/m^2^; species in (b) are *A*, *A. trapezoides*; *H*, *Heteroporodrilus* spp.; *d*, *Microscolex dubius*; *M*, *Megascolecedes* spp; *O*, *other species* (unidentified). Both *Heteroporodrilus* and *Megascolecedes* spp. are native species.

Earthworms were not found in autumn, but were found in variable numbers during the other three seasons. The mean abundance for the whole site doubled from 10.2 m^–2^ in winter to 21.8 m^–2^ in spring, but then fell by 30% to 14.4 m^–2^ in summer (Fig. 2a). The grassland sward consistently had more earthworms than the woodland, while Plantation-06 had the lowest number of earthworms amongst all the vegetation covers. Differences in the earthworm density amongst the vegetation covers were largest and only significant in spring, when the density was in the order Plantation-04 > grassland > woodland > Plantation-06. These differences were marginal in winter and summer despite the population density being at least 25% higher in the grassland sward than in the woodland. The number of species did not exceed three in any of the vegetation covers with fewer species found in summer than in the other season (Fig. 2a).

Only the grassland showed as much species diversity as the woodland, even though native species were found only in the woodland. In winter *Microscolex dubius* accounted for at least 58% of earthworms found in all the four vegetation covers, and were the only species found in the two plantations (Fig. 2b). Unidentified species accounted for about 30% of the earthworms in the woodland and the grassland with balance made up by a native *Megascoleceide* species in the woodland (15%) and *A. trapezoides* (10%) in the grassland. Except for Plantation-06 that contained only *A. trapezoides*, unidentified species constituted between 35% and 70% of the earthworms found in the other vegetation covers in winter (Fig. 2c). In winter a native species *Heteroporodrilus* species accounted for about 8% of the population in the woodland, while *A. trapezoides* accounted for 12% of earthworms in the grassland. In summer, except the woodland in which 18% of the earthworms were of unidentified species, the worm populations were made up entirely of *Microscolex dubius* in all the four vegetation covers (Fig. 2d).

**Fig. 3:**
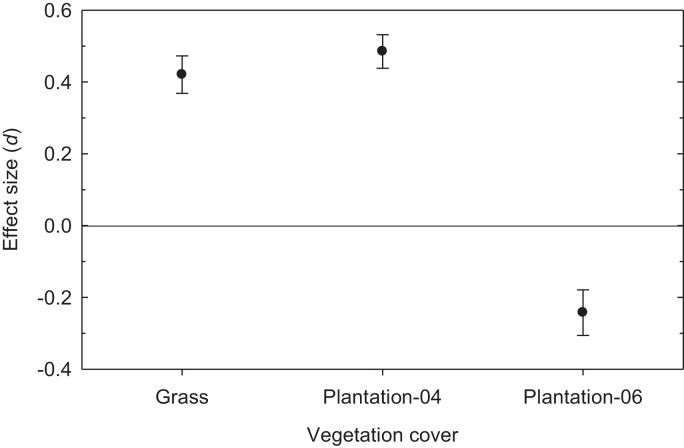
Mean effect size (±95% confidential intervals) for earthworm abundance in the three vegetation covers in spring of 2010 at Castlereagh, Australia. The woodland provides the baseline (zero line) data.

The analysis of effect size (*d*) using abundance data in spring showed significant improvements in earthworm populations with grassland and Plantation-04, but a significant reduction in Plantation-06, relative to the woodland (Fig. 3).

### 3.2 Soil type influence on exotic earthworm species

**Fig. 4:**
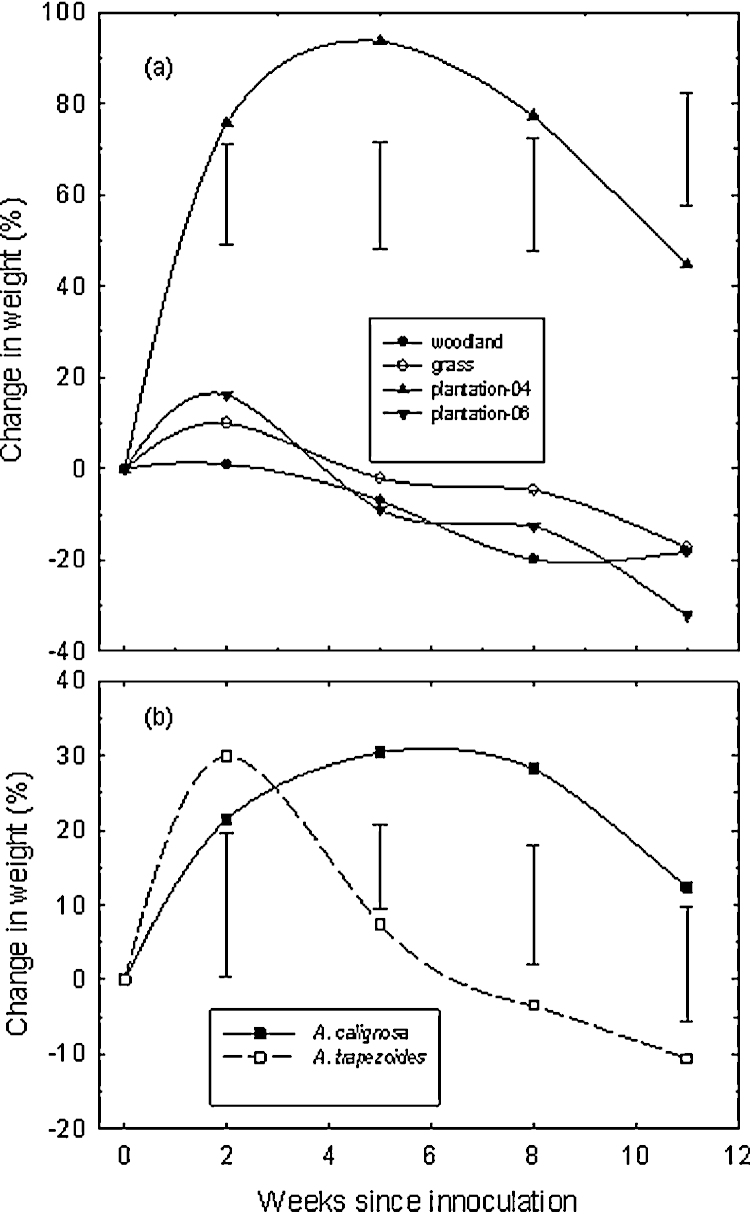
Trends observed in the changes in the mean weights of earthworms due to differences in the vegetation covers (a) and worm species (b). The capped bars are the least significance difference at *p* ≤ 0.05. The mean weight for individual earthworm at the beginning of the experiment was 0.41 ± 0.07 g for *A. caliginosa* and 0.66 ± 0.04 g for *A. trapezoides*.

Survival rate for the two earthworm species was similar in the four soils (Table 3); survival averaged 87 ± 6%. Earthworm biomass increased in all the four soils during the first two weeks of inoculation, more so in Plantation-04 (Fig. 4a). The earthworms continued to grow in Plantation-04 for another three weeks until the 5th week before declining. These initial weight gains in both earthworms were up to 90% in the soil from Plantation-04 compared with <20% in the other three soils. By the end of 11 weeks of incubation, the earthworm species were at least 20% lighter than their starting weights except in Plantation-04 in which the earthworms had a 42% net gain in weight. Changes in weight were more rapid in *A. trapezoides* than in *A. caliginosa*, whereas the latter posted a net gain in weight of 12%, *A. trapezoides* lost 10% of its weight, relative to their initial weights (Fig. 4b).

More cocoons were produced by earthworms in the soil from Plantation-04 than in soils from either grassland or woodland, while none was produced in soil from Plantation-06, after 11 weeks of inoculation (Table 3). During the same period, the earthworms in woodland soil produced the most casts, which was more than twice that produced in soil from Plantation-04, and several factors larger (>5) than cast produced in soils from the grassland or Plantation-06 (>8). Many more burrows were created in the soil from Plantation-04 or from grassland than in the soil from either Plantation-06 or woodland. *A. trapezoides* produced more cocoons and burrows than *A. calignosa* during the 11 weeks of the trial (Table 3).

At the end of the 11th week, the pH was similar in soils from grassland and Plantation-04 (6.4 ± 0.2) compared with soils from woodland or Plantation-06 (6.0 ± 0.1). Also the soil EC was higher under the two plantations than under the grassland and the woodland (Table 3). The mean weight of earthworms was significantly correlated with pH (*r* = 0.91), EC (*r* = 0.93), clay content (*r* = 0.75), sand (*r* = −0.99) and phosphorus content (*r* = −0.53), of the soil. As a result of these correlations, influences of variable EC and sand content on earthworms were explored further.

### 3.3 Soil textural and nutritional influence on exotic earthworms

Amending the clay soil from Plantation-04 with sand and/or organic matter did not change the pH, which averaged 5.6 ± 0.1 (data not presented), but addition of organic matter significantly increased salinity (Table 4). The earthworms lost weight in unamended soil over the 4-week period, but gained weight in the amended soils. *A. trapezoides* lost more weight in unamended soil, and gained proportionally less weight in the amended soil, than *A. gracilis*. While no casts were produced by earthworms in the unamended soil, those in amended soil each produced about 1.0 g of cast over the 4-week period. Amendment of the clayey soil from Plantation-04 with sand reduced the bulk density (Table 4).

Application of fertilizer to the loamy sand from the woodland reduced pH to 5.0 ± 0.1 from 5.4 ± 0.1 in the untreated soil; it also increased salinity (Table 4). While *A. gracilis* did not lose weight in amended soil, it lost 12% of its mass in the untreated soil. By contrast, *A. trapezoides* maintained its weight in the unfertilized soil but gained 14% in weight with fertilizer application.

## 4 Discussion

### 4.1 Seasonal dynamics in earthworm populations

Seasonal abundance and diversity in earthworm populations were reflections of weather conditions and characteristics of the vegetation covers. Earthworm catches are sensitive to moisture and temperature conditions of the soil. The absence of catches in autumn was consistent with the relatively high soil temperature (>20 °C) at this time (Table 2), which was outside the optimum range of 10–15 °C for releasing the earthworms from dormancy and thrive (Baker and Whitby, 2003; Lee, 1985). The autumn in 2010 was also relatively dry with rainfall events being few and far between (Fig. 1) and this condition was only slightly alleviated by winter (June–August), which along with exceptionally low minimum air temperature, must have constrained earthworm hatches at this time. By spring however, the confluence of low temperatures and several rainfall events provided cool and moist soil conditions resulting in most earthworms being found at this time (Fig. 2a). These favourable conditions largely persisted into early summer in December, such that there was a mean decline of only 30% in earthworm abundance compared with spring.

Both the grassland and Plantation-04 consistently maintained parity or out-performed the woodland in terms of earthworm abundance, but this could not be associated with soil fertility indices presented in Table 1. The soil C was lower in Plantation-6 than in either grassland or the woodland, while the woodland had lower N and CEC than all the other vegetation covers. Worm abundance was not even consistent with the differences in the C:N ratios of the soil that determines digestibility of surface litter as sources of nutrients (Ndegwa and Thompson, 2000). It was only in the woodland soil that the C:N of 22.5 (Table 1) was close to the optimum of 25 for earthworm viability and function; while the values for the soils in the grassland (11.7) and Plantation-04 (6.2) were far from ideal. This inconsistency between fertility indices and earthworm abundance across the four vegetation covers, however, was consistent with the earlier finding by Staaf (1987) that abundance and species composition of earthworms were not explained by the concentrations of lignin, N, or P or their combinations in the litter.

Differences amongst the vegetation covers in the earthworm abundance were also not consistent with the trends in the pH or salinity of the soil. Neither could they be associated with concentrations of heavy metals in the soils since high concentrations of Cr, Cu, Ni, Pb and Zn that are known to be injurious to earthworms (Muir et al., 2007; Spurgeon and Hopkin, 1999) were higher in the three revegetated covers than in the woodland (Table 1). For instance, the concentration of Zn, which is known to be especially toxic to earthworms (Muir et al., 2007; Spurgeon, Svendsen, Rimmer, Hopkin, & Weeks, 2000), was larger by a factor of 3–4 in the grassland soil than in the soils of the two plantations and the woodland.

The most likely explanation for differences in the abundance of earthworms was the age of the vegetation covers. Since disruption of the habitat is the primary threat to earthworm viability, it is logical that the length of post rehabilitation period has direct bearing on the degree of recovery in the earthworm community. While the grassland and Plantation-04 exceeded woodland in restoring earthworm numbers, Plantation-06 had yet to attain this status due to its juvenile age (Fig. 3). This was consistent with an observation by Scullion et al. (1988) that a minimum of 5 years was needed for earthworms to recover on rehabilitated mine site. Without any significant perturbation no measurable difference in earthworm populations were found between grassland, woodland and forest in the savannah (Moïse, Seydou, Kolo, & Mamadou, 2011). However, species diversity was still poor in the plantations and grassland neither of which contained native earthworm species (Fig. 2b–d). The dominance of *M. dubius* and *A. trapezoides*, especially the former, in all the vegetation covers at this site was consistent with both species being widely distributed on cultivated landscapes in Australia, where they have largely displaced the native species (Chan and Barchia, 2007; Chan and Heenan, 2006; Hendrix et al., 2006), and on contaminated lands elsewhere (Spurgeon and Hopkin, 1999).

### 4.2 Vegetation type and earthworm viability

The absence of clear correlation between soil properties and earthworm population amongst the four vegetation covers could be the result of large variability in soil properties commonly encountered in the field. Such variability is often not apparent in soil properties obtained from bulked samples such as those presented in Table 1, but could be large enough to create discrete niches that are conducive for various macrofauna, including earthworms. Bulked samples, however, are still appropriate for testing how earthworm growth and reproduction potential respond to chemical and nutritive properties of individual soil type. It was therefore possible to ascribe the significant weight gains and retention by earthworms in the soil from Plantation-04 (Fig. 4a) to the higher fertility of this soil in which P and EC were higher than in the other soils (Table 1); these were also reflected in the differences in EC amongst the potted soils from the four vegetation covers following 11 weeks of inoculation (Table 3). Indeed we found significant correlation between weight gain with EC (*r* = 0.81) as with pH (*r* = 0.83). Although land preparation and establishment procedures were largely similar for both plantations and the grassland, substantial amounts of nutrients would have leached out over the past 16 years from the grassland sward; while Plantation-06 received less preparation, including fertilization, at establishment, and also lacked topsoil for several years afterwards (Yunusa et al., 2010).

Reasons for the subsequent loss in weight by the earthworms were not quite clear. It is possible, however, that key nutrients that were initially readily available were quickly exhausted, but less rapidly in the more fertile soil from Plantation-04. The large amounts of casts produced in the other three soils (Table 3) suggests greater foraging for nutrients that is often associated with increased burrowing, but known to impair weight gain (Bottinelli, Henry-des-Tureaux, Hallaire, Mathieu, & Benard, 2010). This was also consistent with the positive correlation between the mean weight of the earthworms and EC (*r* = 0.93) as a measure of soluble mineral nutrients in the soil, and with the weight gained by the earthworms when the woodland soil was amended with fertilizer or organic matter or both (Table 4). However, the subsequent weight loss could also be the result of the earthworms attaining maturity and onset of reproductive phase and cast formation. This critical phase could have been attained earlier in *A. trapezoides* than in *A. caliginosa*, consistent with the earlier onset of weight loss in the former (Fig. 4b). Thus *A. trapezoides* produced 10 times more cocoons than *A. caliginosa* (Table 3), with the former producing more than its initial mean weight in cocoons during the 11-week period. A response that could be associated with preference of *A*. *trapezoides* for compost and animal manure (used as food source in this study) compared with *A. gracilis* natural preference for decaying plant matter (Barois, 1992). Also, increases in earthworm weight in amended clayey soil could partly be due to the reductions in the bulk density as found in an earlier study (Hendrix et al., 2006; Reinecke, 1983).

In the present study Plantation-04 effectively restored the earthworm population to preclearance levels. This vegetation system was also found in an earlier study to be quite effective in restoring hydrologic function to the site (Yunusa et al., 2010, 2012) and will therefore be preferred over the grassland cover for rehabilitating this and similar sites.

## 5 Conclusions

We initiated this study with the expectation that the choice of vegetation cover in rehabilitating waste disposal or mining sites would produce differential outcomes in terms of soil biota. Our data, however, suggest that the differences in soil properties created by the contrasting vegetation covers were not large enough to impact earthworm abundance. Recovery of species diversity in the reclaimed vegetation covers was still well below that found in the old growth woodland. The grassland was devoid of native species even after 16 years suggesting that much longer time is needed for the return of these species to such environment. All the earthworms recovered in the woodland were endogeic, even the native species, and thus belong to the same functional group as the exotic species that dominated in the grassland and the plantations. Therefore the functions of earthworms in maintaining soil structure and nutrient cycling (Bottinelli et al., 2010; Lee, 1985) would have been largely restored to preclearance levels within six years of revegetation (Table 1); this process would not be any slower in grassland that had even a more rapid establishment than woody species. Ecological restoration of disrupted landscapes having duplex soil, such as used in this study, can be enhanced by mixing fine particles from the mineral clayey subsoil with the coarse particles from the loamy topsoil that is relatively high in organic carbon to promote earthworm establishment and growth, and general belowground biota.

## Figures and Tables

**Table 1:** Soil properties for the topsoil (surface 0.3 m) under the four vegetation types at Castlereagh, New South Wales, Australia.

Properties	Woodland	Grass	Plantation-04	Plantation-06
*Particle size distribution (%)*				
Gravel	5	27	52	30
Sand	92	49	31	54
Silt	3	6	5	2
Clay	0	18	12	14

*Basic chemical property*				
Soil pH (1:5 H_2_O)	5.8	6.8	5.8	6.7
Soil salinity (dS m^−1^)	0.04	0.11	0.17	0.13
Bulk density (Mg/m^3^)	1.20	1.48	1.32	1.28
Total porosity (%)	55	45	51	52
Total C %	0.90	0.82	0.31	0.23
Total N %	0.04	0.07	0.05	0.05
C:N	22.5	11.7	6.2	4.6
CEC (cmol+/kg)	2.27	9.62	9.82	10.22

*Concentration of selected trace metals (mg/kg)*				
Arsenic	7	20	9	19
Lead	12	38	17	29
Chromium	40	87	44	76
Copper	4	21	17	13
Manganese	25	116	19	33
Zinc	7	26	18	26

**Table 2:** Predicted mean soil temperature (°C) at 0.2 m depth under the various vegetation covers at the times of sampling in 2010.

Vegetation covers	Autumn	Winter	Spring	Summer
Woodland	23.0	16.7	18.2	19.2
Grass	24.1	17.8	19.1	20.2
Plantation-04	21.5	14.9	16.3	19.7
Plantation-06	23.6	17.3	17.8	19.5

Prediction was obtained with model of Paul et al. (2004).

**Table 3:** Characteristics of the earthworms inoculated in, and electrical conductivity (EC) of, the soils collected from the vegetation covers measured 11 weeks after inoculation.

Treatments	Survival (%)	Cocoons/worm (g)	Casts/worm (g)	Burrows (no./m^2^)	EC (dS m^−1^)
*Vegetation covers*					
Woodland	85	0.13b	1.65a	219b	0.03c
Grass	89	0.13b	0.34c	365a	0.06bc
Plantation-04	87	1.25a	0.69bc	390a	0.13a
Plantation-06	83	0.00c	0.19c	244b	0.09b
SE	6.9	0.22	0.30	28.9	0.01

*Species effects*					
*A. caliginosa*	81	0.06b	0.82	349a	0.08
*A. trapezoides*	90	0.69a	0.62	260b	0.10
SE	25.3	0.27	0.21	20.4	0.02

Means followed with different letter(s) are statistically different at *p* < 0.05.

**Table 4:** Responses in selected soil properties and in the growth of earthworm species to amendments of soils from the woodland and Plantation-04 measured four weeks after inoculation.

Soil amendments	Electrical conductivity (dS m^−1^)	Bulk density (Mg/m^3^)	Initial weight/final weight^a^	
			*A. gracilis*	*A. trapezoides*
*Textural alterations of Plantation-04 soil*				
Control	0.15b	1.33a	0.90b	0.63b
Sand	0.15b	1.02b	4.03a	1.23a
Organic matter	0.21a	1.27ab	3.58a	1.23a
Sand + organic matter	0.22a	1.11b	3.56a	1.27a
SE	0.01	0.12	0.71	0.15

*Nutritional alterations of woodland soil*				
Control	0.03	1.22	0.88b	0.99b
*Fertiliser*	0.07	1.23	0.99a	1.14a
SE	0.02	0.18	0.07	0.03

Means followed with different letter(s) are statistically different at *p* < 0.05.

a Intial weights were 0.90 g for *A. gracilis* and 0.25 g for *A. trapezoides*.

## Appendix A Supplementary data

The following KML file contains the Google map of the most important is as described in this article.

## References

[bib0005] Baker G.H., Whitby W.A. (2003). Soil pH preferences and the influences of soil type and temperature on the survival and growth of *Aporrectodea longa* (Lumbricidae): The 7th international symposium on earthworm ecology Cardiff Wales 2002. Pedobiology.

[bib0155] Barois I. (1992). Mucus production and microbial activity in the gut of two species of Amynthas (Megascolecidae) from cold and warm tropical climates. Soil Biology and Biochemistry.

[bib0010] Blakemore R. (2008). Cosmopolitan earthworms – An eco-taxonomic guide to the peregrine species of the world.

[bib0015] Bottinelli N., Henry-des-Tureaux T., Hallaire V., Mathieu J., Benard Y. (2010). Earthworms accelerate soil porosity turnover under watering conditions. Geoderma.

[bib0020] Boyer S., Wratten S.D. (2010). The potential of earthworms to restore ecosystem services after opencast mining – A review. Basic & Applied Ecology.

[bib0025] Bradshaw A.D. (1984). Ecological principles and land reclamation practice. Landscape & Urban Planning.

[bib0030] Bradshaw A.D. (2000). The use of natural processes in reclamation — Advantages and difficulties. Landscape & Urban Planning.

[bib0035] Chan K.Y., Barchia I. (2007). Soil compaction controls the abundance, biomass and distribution of earthworms in a single dairy farm in south-eastern Australia. Soil & Tillage Research.

[bib0040] Chan K.Y., Heenan D.P. (2006). Earthworm population dynamics under conservation tillage systems in south-eastern Australia. Soil Research.

[bib0045] FAO, 1974. *Key to the FAO Soil Units*. http://www.fao.org/nr/land/soils/key-to-the-fao-soil-units-1974/en/. Accessed: 02.09.2011.

[bib0050] Gollan J.R., Lobry de Bruyn L., Reid N., Smith D., Wilkie L. (2010). Can ants be used as ecological indicators of restoration progress in dynamic environments? A case study in a revegetated riparian zone. Ecological Indicators.

[bib0055] Grant C.D., Campbell C.J., Charnock N.R. (2002). Selection of species suitable for derelict mine site rehabilitation in New South Wales, Australia. Water Air & Soil Pollution.

[bib0060] Gurevitch J., Hedges L.V., Scheiner S.M., Gurevitch J. (1993). Meta-analysis: Combining the results of independent experiments. Design and analysis of ecological experiments.

[bib0065] Hendrix P.F., Baker G.H., Callaham M.A., Damoff G.A., Fragoso C., Gonza’ lez G. (2006). Invasion of exotic earthworms into ecosystems inhabited by native earthworms. Biological Invasions.

[bib0070] Itakura T., Airey D.W., Dobrolot J.Y.M. (2005). Geotechnical characteristics of alluvial soils used to contain industrial liquid wastes. Bulletin of Engineering, Geology & Environment.

[bib0075] Lee K.E. (1985). Earthworms: Their ecology and relationships with soils and land use.

[bib0080] Moïse E.N., Seydou T., Kolo Y., Mamadou D. (2011). Plant community influences on earthworms in Lamto savannahs (Côte d’Ivoire). Journal of Applied Bioscience.

[bib0085] Muir M.A., Yunusa I.A.M., Burchett M.D., Lawrie R., Chan K.Y., Manoharan V. (2007). Short-term responses of two contrasting species of earthworms in an agricultural soil amended with coal-fly ash. Soil Biology & Biochemistry.

[bib0090] Ndegwa P.M., Thompson S.A. (2000). Effects of C-to-N ratio on vermicomposting of biosolids. Bioresource Technology.

[bib0095] Paul K.I., Polglase P.J., Smethurst P.J., O’Connell A.M., Carlyle C.J., Khanna P.K. (2004). Soil temperature under forests: A simple model for predicting soil temperature under a range of forest types. Agricultural and Forest Meteorology.

[bib0100] Reinecke A.J., Satchell J.E. (1983). The ecology of earthworms in Southern Africa. Earthworm ecology: From Darwin to Vermiculture.

[bib0105] Richardson J.A., Burn I., Craig G. (1987). Long-term prospects for farm grassland and woodland on reclaimed coal spoil sites. Landscape & Urban Planning.

[bib0110] Scullion J., Ramshaw G.A., Mohammed A.R.A. (1988). Changes in earthworm populations following cultivation of undisturbed and former opencast coal-mining land. Agricultural Ecosystem & Environment.

[bib0115] Smith R.G., McSwiney C.P., Grandy A.S., Suwanwaree P., Snider R.M., Robertson G.P. (2008). Diversity and abundance of earthworms across an agricultural land-use intensity gradient. Soil & Tillage Research.

[bib0120] Spurgeon D.J., Hopkin S.P. (1999). Seasonal variation in the abundance, biomass and biodiversity of earthworms in soils contaminated with metal emissions from a primary smelting works. Journal of Applied Ecology.

[bib0125] Spurgeon D.J., Svendsen C., Rimmer V.R., Hopkin S.P., Weeks J.M. (2000). Relative sensitivity of life-cycle and biomarker responses in four earthworm species exposed to zinc. Environmental Toxicology & Chemistry.

[bib0130] Staaf H. (1987). Foliage litter turnover and earthworm populations in three beech forests of contrasting soil and vegetation types. Oecologia.

[bib0135] Weir B.J., Fulton I., Menzies N.W. (2006). Revegetation strategies for bauxite refinery residue: A case study of Alcan Gove in Northern Territory, Australia. Environmental Management.

[bib0140] Yunusa I.A.M., Braun M., Lawrie R. (2009). Amendment of soil with coal fly ash modified the burrowing habits of two earthworm species. Applied Soil Ecology.

[bib0145] Yunusa I.A.M., Zeppel M.J.B., Fuentes S., Macinnis-Ng C.M.O., Palmer A.R., Eamus D. (2010). An assessment of the water budget for contrasting vegetation covers associated with waste management. Hydrological Processes.

[bib0150] Yunusa I.A.M., Zolfaghar S., Zeppel M.J.B., Li Z., Palmer A.R., Eamus D. (2012). Fine root biomass and its relationship to evapotranspiration in woody and grassland vegetation covers for ecological restoration of waste storage and mining landscapes. Ecosystems.

